# Serum amyloid beta 42 levels correlated with metabolic syndrome and its components

**DOI:** 10.3389/fendo.2024.1278477

**Published:** 2024-02-08

**Authors:** Kecheng Li, Xiaoli Zhou, Youren Liu, Dongyu Li, Yinyin Li, Ting Zhang, Chunyan Fu, Lin Li, Yang Hu, Li Jiang

**Affiliations:** ^1^ Department of Laboratory Medicine, Sichuan Provincial People’s Hospital, School of Medicine, University of Electronic Science and Technology of China, Chengdu, Sichuan, China; ^2^ Sichuan Provincial Key Laboratory for Human Disease Gene Study, Sichuan Provincial People’s Hospital, University of Electronic Science and Technology of China, Chengdu, Sichuan, China; ^3^ Department of Health Management, Sichuan Provincial People’s Hospital, University of Electronic Science and Technology of China, Chengdu, China; ^4^ Department of Gastrointestinal Surgery, Sichuan Provincial People’s Hospital, University of Electronic Science and Technology of China, Chengdu, Sichuan, China

**Keywords:** metabolic syndrome, serum amyloid beta 42 (Aβ42), body mass index (BMI), blood lipids, blood pressure, Alzheimer’s disease (AD)

## Abstract

**Introduction:**

Beta-amyloid accumulation in the brain appears to be a key initiating event in Alzheimer’s disease (AD), and factors associated with increased deposition of beta-amyloid are of great interest. Enhanced deposition of amyloid-β peptides is due to an imbalance between their production and elimination. Previous studies show that diminished levels of CSF amyloid beta 42 (Aβ42) is a biomarker in AD; however, the role of serum Aβ42 in AD is contradictory. BMI and obesity have been reported to be related to increased serum Aβ42 levels. Therefore, we aimed to investigate the relation between metabolic syndrome (MetS), its clinical measures (abdominal obesity, high glucose, high triglyceride, low high-density lipoprotein cholesterol level, and hypertension), and serum Aβ42 levels.

**Methods:**

A total of 1261 subjects, aged 18–89 years in Chengdu, China, were enrolled from January 2020 to January 2021 to explore the correlation of serum Aβ42 levels with body mass index (BMI), blood lipids, and blood pressure. Furthermore, as the risk of MetS is closely related to age, 1,212 participants (*N* = 49 with age ≥ 80 years old were excluded) were analyzed for the correlation of serum Aβ42 level and MetS clinical measures.

**Results:**

The results showed that log-transformed serum Aβ42 level was positively correlated with BMI (*R* = 0.29; *p* < 0.001), log-transformed triglyceride (*R* = 0.14; *p* < 0.001), and diastolic blood pressure (DBP) (*R* = 0.12; *p* < 0.001) and negatively correlated with high-density lipoprotein (HDL-c) (*R* = −0.18; *p* < 0.001). After adjusting for age, sex, and other covariates, elevated serum Aβ42 level was correlated with higher values of BMI (βmodel1 = 2.694, βmodel2 = 2.703) and DBP (βmodel1 = 0.541, βmodel2 = 0.546) but a lower level of HDL-c (βmodel2 = −1.741). Furthermore, serum Aβ42 level was positively correlated with MetS and its clinical measures, including BMI and DBP, and negatively correlated with HDL-c level in the Han Chinese population. However, the level of serum Aβ42 did not show a significant correlation with high glucose or high triglyceride.

**Discussion:**

These observations indicate that MetS and its components are associated with higher levels of serum Aβ42 and hence limit the potential of serum Aβ42 as a suitable diagnostic biomarker for AD. As such, we recommend serum Aβ42 serve as a direct risk biomarker for MetS rather than for AD.

## 1 Introduction

The accumulation of amyloid-β (Aβ) peptides (mainly Aβ42 and Aβ40) in the brain parenchyma and cerebral vasculature is a major hallmark of AD pathogenesis (1, 2). The amyloid hypothesis reveals that amyloid precursor protein (APP) is cleaved into pathological forms of Aβ by β- and γ-secretase enzymes, driving the imbalance between Aβ production and clearance (3). Aβ42 is the most abundant protein in amyloid plaques due to its higher rate of fibrillization and insolubility. Aβ42 in the cerebrospinal fluid (CSF) has been established as a reliable biomarker to support an AD diagnosis. Additionally, studies have shown that changes in CSF Aβ are greater than those in plasma because CSF is in direct contact with the brain and only a small fraction of brain proteins reaches the bloodstream (4, 5). Hence, CSF Aβ42, plasma-based two fractions of β amyloid peptide ratio (Aβ42/40), and phosphorylated tau (p-tau) are considered promising prospective biomarkers for AD diagnosis and progression (6, 7).

The imbalance between the production and clearance of Aβ, which occurs not only in the brain but also in the periphery, is considered an initial factor in AD (8). An increasing number of studies support the hypothesis that systemic abnormalities (circadian rhythm, oxidative stress, metabolic syndrome, etc.) are risk factors for AD development, especially metabolic syndrome (MetS) and its individual components, including abdominal obesity, high glucose, high triglyceride, low high-density lipoprotein cholesterol levels, or hypertension (8–13). MetS is also associated with an increased risk of developing cardiovascular disease, AD, and dementia (3, 10, 14–17). Recently, a study also showed that MetS causes a fast decline in cognitive performance and stimulates Aβ42 production in the brain (18). The prevalence of MetS reached approximately 25% of the global population in 2018 and increased every year (19).

Evidence indicates that central obesity plays a central role in the development of the MetS and appears to precede the appearance of the other MetS components (14). Several previous meta-analyses implied that midlife obesity was a potentially modifiable risk factor for dementia and AD, but this is still uncertain with rather heterogeneous results (20–23). Recent studies revealed APP is highly expressed in adipose tissue and upregulated in obesity (24). The concentration of Aβ in blood was significantly increased in both mouse models and obese individuals (25, 26). In addition, there has been evidence showing plasma Aβ42 level was positively correlated with BMI in small groups of nondemented adults and children (27–30). A prospective study indicated an increase over 5 years of HDL-c was a negative predictor for the decrease of plasma Aβ42 levels (31), while another study showed a positive correlation of plasma Aβ42 levels with HDL (32). Additionally, abnormal blood pressure is tightly associated with dysregulated lipid metabolism (33). An animal study revealed that Aβ-induced hypertension can be an early pathophysiologic consequence of AD processes (34). One cross-sectional study also revealed that many factors influence the association between plasma Aβ42 levels and AD cognitive impairment, and they proposed that plasma Aβ42 may be a peripheral biomarker for AD screening in the Chinese elderly population, but it is necessary to establish standardized detection methods and establish different demarcation criteria for various influencing factors (35).

Therefore, whether and how serum Aβ42 level is directly related to MetS and its components in the Han population on a large scale is still vague. We hypothesize that the concentration of Aβ42 in the periphery is a biomarker of MetS and its components, independent from AD. To test this, we conducted a cross-sectional study in a regular health check-up population to analyze the association of serum Aβ42 level with MetS and its clinical measures, including abdominal obesity, high glucose, high triglyceride, low high-density lipoprotein cholesterol levels, and hypertension.

## 2 Methods

### 2.1 Participants

**Figure 1:**
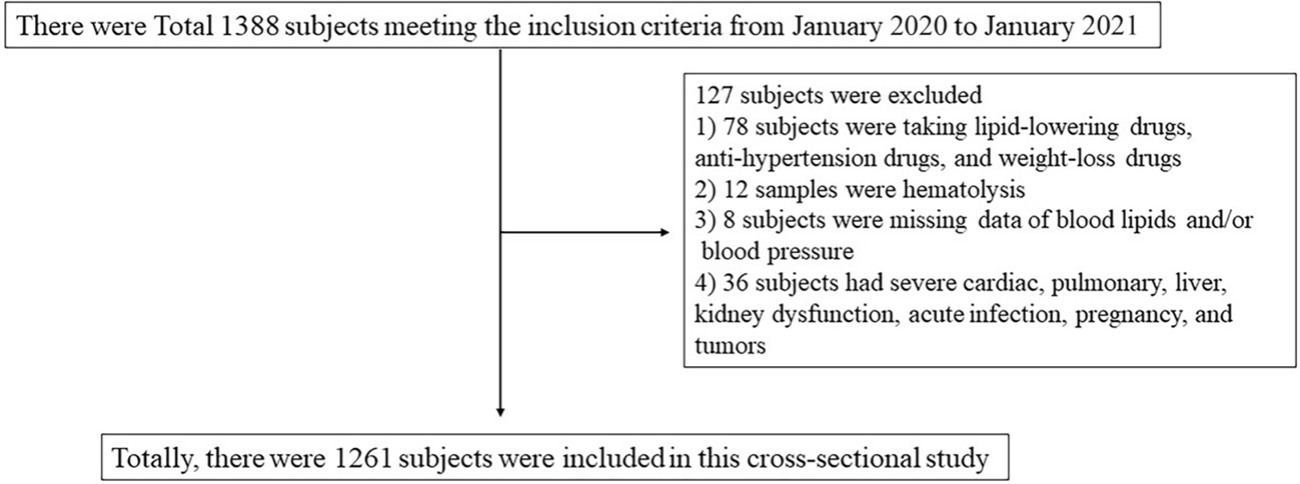
Flow chart of participant screening.

We enrolled a total of 1,261 participants between the ages of 18–89 years from a regular health check-up population in the Sichuan Academy of Medical Sciences and Sichuan Provincial People’s Hospital between January 2020 to January 2021 (**Figure 1**). Participants were excluded by diagnoses of CAD, renal disease, autoimmune disease, hypersensitivity, gastrointestinal disease, pulmonary disease, cancer, acute illness, or hospitalization within 15 days. This study was approved by the Ethics Committee of the Sichuan Academy of Medical Sciences and Sichuan Provincial People’s Hospital (2020No.281). All subjects provided written informed consent prior to participation.

### 2.2 Diagnostic criteria of obesity, hypertension, and dyslipidemia

According to the health criteria WS/T 428-2013 issued by the National Health Commission of the People’s Republic of China, subjects with BMI ≥ 28, 24 < BMI < 28, and BMI ≤ 24 are defined as obese, overweight, and normal weight, respectively. According to the 2020 International Society of Hypertension global hypertension practice guidelines, a subject with systolic blood pressure (SBP) ≥ 140 mmHg and/or diastolic blood pressure (DBP) ≥ 90 mmHg is defined as hypertensive (36). According to the Chinese guidelines for the prevention and treatment of dyslipidemia, a subject with total cholesterol (TC) ≥ 5.18 mmol/L and/or triglyceride (TG) ≥ 1.70 mmol/L and/or low-density lipoprotein cholesterol (LDL-c) ≥ 3.37 mmol/L and/or HDL-c ≤ 1.04 mmol/L is defined as having dyslipidemia (37).

### 2.3 Diagnostic criteria of metabolic syndrome

In this study, the modified ATP III criteria were applied in the diagnosis of MetS, which requires the presence of at least three abnormal findings out of five factors (38): (i) Abdominal density as defined by waist circumference ≥ 90 cm and ≥ 80 cm in men and women, respectively according to the Asian World Health Organization criteria.; (ii) TG ≥ 1.7 mmol/L; (iii) HDL-c < 1.03 mmol/L and < 1.29 mmol/L in men and women, respectively; (iv) SBP ≥ 130 mmHg or DBP ≥ 85 mmHg; and (v) glucose (GLU) ≥ 5.6 mmol/L as impaired fasting glucose (IFG).

### 2.4 General information collection and blood pressure measurement

All participants were required to fill out a questionnaire to collect general information, including weight, height, waist circumference, etc. A mercury sphygmomanometer was applied on the right arm (with a regular adult cuff) of each participant after having rested for 5 min in a seated position to measure blood pressure (SBP and DBP) before blood sample collection.

### 2.5 Blood collection and biochemical analysis

Fasting blood was collected into serum separator tubes (BD, Franklin Lakes, NJ, USA) with a standard venipuncture technique in the morning. Serum was separated from the blood samples by centrifugation at 1,000×*g* for 20 min immediately after clotting and stored in an aliquot at −80°C until laboratory analysis of Aβ42 and other relevant biomarkers in this study. Biochemical analysis including serum TC, TG, HDL-c, LDL-c, and GLU was performed using the Abbott ARCHITECT c16000 clinical chemistry system (Abbott Co., Chicago, USA) in the Department of Laboratory Medicine, Sichuan Academy of Medical Sciences, and Sichuan Provincial People’s Hospital.

### 2.6 Measurement of serum Aβ42

The concentration of serum Aβ42 was measured by a double-antibody sandwich method with a commercial kit (Mlbio Co., Shanghai, China) according to its manufacturer’s instructions. In detail, 50 μL of serum sample or Standard were added in duplicate to the appropriate well of the 96-well microtiter plate that had been precoated with antihuman Aβ1–42 capture antibody, and subsequently, 100 μL of HRP-conjugated detecting antibody was added to each well except the blank well. The microtiter plate was incubated for 60 min at 37°C and then manually wash with wash solution (1×) four times. After that, 50 μL of substrate A and 50 μL of substrate B were added to each well of the microtiter plate and incubated for 15 min in the dark at 37°C. After adding 50 μL of stop solution to each well, the O.D. at OD450 was measured with a microtiter plate reader (Bio-Rad, Californie, USA) within 15 min. The mean O.D. value of two wells was calculated for each standard and sample and subtracted by the mean value of the blank wells. The standard curve was generated by plotting the O.D. of the six standards on the vertical (*x*)-axis versus the corresponding concentration of Aβ42 (240 pg/mL, 120 pg/mL, 60 pg/mL, 30 pg/mL, 15 pg/mL, or 7.5 pg/mL) on the horizontal (*y*)-axis. The concentration of Aβ42 in each sample was then determined by plotting its O.D. in the standard curve.

### 2.7 Statistical analysis

The skewness, kurtosis, and P-P plots were used to test the distribution of each covariate. Age, TC, HDL-c, LDL-c, SBP, DBP, BMI, hip circumference, and waist circumference conformed to a normal distribution, and these covariates were expressed as mean (SD). Serum Aβ42 levels, TG, and GLU did not conform to the normal distribution. Hence, they were expressed as median (interquartile range) and were log-transformed for further analysis. The participants were divided into different groups according to their BMI, blood lipids, and blood pressure. The covariates were compared between different groups by unpaired Student’s *t*-test, Mann–Whitney *U* test, and Kruskal–Wallis test. Categorical variables were expressed as numbers (percentage) and were compared by *χ* ^2^ tests. Pearson correlation was performed to explore the linear trend between log-transformed serum Aβ42 levels and BMI, blood lipids, and blood pressure. In addition, multiple linear regression analysis was performed to further explore their potential relationships, with a variance inflation factor (VIF) higher than 10 considered colinear. All these statistical analyses were performed by SPSS 22.0 (SPSS Inc., Chicago, IL, USA), and a *p*-value of less than 0.05 was considered statistically significant.

## 3 Results

### 3.1 Clinical characteristics of the participants

A total of 1,261 participants were analyzed for correlation between serum Aβ42 levels and BMI, blood lipids, and blood pressure, as shown in **Tables 1**–**4**. The anthropometric and metabolic characteristics of the subjects are summarized in each table. As the risk of MetS is closely associated with age, we tested this association in adults across ages (39, 40). We then classified the participants into three age groups, young (18–29 years), middle-aged (30–64 years), and old (65–79 years) adults. Therefore, 1,212 participants were analyzed including 459 individuals with MetS diagnosed by the modified ATP III criteria (**Table 5**).

**Table 1:** Comparison of serum Aβ42 levels among participants divided by BMI.

	Normal weight (*n* = 348)	Overweight (*n* = 414)	Obesity (*n* = 499)	*p*-value
Age (years)	50.93 ± 17.68	52.40 ± 14.35	52.00 ± 15.11	0.433
Male (*n* (%))	153 (43.97)	249 (59.90)	274 (54.91)	<0.001
Smoking (*n* (%))	119 (34.19)	121 (29.22)	153 (30.67)	0.321
Drinking (*n* (%))	58 (16.67)	76 (18.36)	62 (12.42)	0.110
BMI (kg/m^2^)	21.62 ± 1.66	25.69 ± 1.11	30.09 ± 2.07	<0.001
Waistline (cm)	76.18 ± 7.62	87.55 ± 6.67	96.59 ± 8.09	<0.001
Hip circumference (cm)	91.98 ± 4.42	97.45 ± 4.46	103.93 ± 5.68	<0.001
SBP (mmHg)	123.874 ± 19.68	129.80 ± 17.99	133.82 ± 17.64	<0.001
DBP (mmHg)	72.94 ± 10.64	77.45 ± 10.93	80.08 ± 11.57	<0.001
GLU (mmol/L)	5.03 ± 4.7	5.27 ± 4.86	5.38 ± 4.96	<0.001
LDL-c (mmol/L)	2.62 ± 0.75	2.85 ± 0.79	2.85 ± 0.75	<0.001
HDL-c (mmol/L)	1.50 ± 0.34	1.32 ± 0.29	1.25 ± 0.26	<0.001
TC (mmol/L)	4.79 ± 0.95	5.00 ± 1.02	4.92 ± 0.94	0.012
TG (mmol/L)	1.12 ± 0.76	1.56 ± 1.05	1.66 ± 1.18	<0.001
ApoE ϵ4 (*n* (%))	46 (13.22)	49 (11.84)	56 (11.22)	0.675
Aβ42 (pg/mL)	36.63 ± 20.10	54.35 ± 33.64	60.24 ± 39.21	<0.001

Aβ, amyloid beta; BMI, body mass index; TC, total cholesterol; TG, triglyceride; HDL-c, high-density lipoprotein; LDL-c, low-density lipoprotein; SBP, systolic blood pressure; DBP, diastolic blood pressure; ApoE, apolipoprotein E. Subjects with BMI ≥ 28, 24 < BMI < 28, and BMI ≤ 24 are defined as obese, overweight, and normal weight, respectively. A subject with at least one allele of ϵ4 is defined as an ApoE ϵ4 carrier.

**Table 2:** Comparison of serum Aβ42 levels among participants divided by TC, TG, LDL-c, and HDL-c.

	High TC (*n* = 474)	Normal TC (*n* = 787)	*p*-value	High TG (*n* = 506)	Normal TG (*n* = 755)	*p*-value	High LDL-c (*n* = 258)	Normal LDL-c (*n* = 1,003)	*p*-value	Low HDL-C (*n* = 196)	Normal HDL-C (*n* = 1,065)	*p*-value
Age (years)	52.87 ± 13.76	51.23 ± 16.65	0.071	51.79 ± 14.54	51.88 ± 16.32	0.925	53.16 ± 13.72	51.51 ± 16.07	0.194	50.71 ± 15.55	52.05 ± 15.64	0.267
Male (*n* (%))	262 (55.27)	414 (52.60)	0.351	328 (64.82)	348 (46.09)	<0.001	153 (59.30)	523 (52.14)	0.036	141 (71.94)	535 (50.23)	<0.001
Smoking (*n* (%))	160 (33.76)	233 (29.61)	0.13	170 (34.78)	223 (28.37)	0.10	86 (33.33)	307 (30.61)	0.41	69 (35.20)	324 (30.42)	0.18
Drinking (*n* (%))	63 (13.29)	133 (16.90)	0.09	69 (13.64)	127 (16.82)	0.15	34 (13.18)	162 (16.15)	0.29	29 (14.80)	167 (15.68)	0.83
BMI (kg/m^2^)	26.44 ± 3.59	26.23 ± 3.98	0.329	27.20 ± 3.38	25.71 ± 4.00	<0.001	26.89 ± 3.72	26.16 ± 3.85	0.176	28.24 ± 3.44	25.95 ± 3.80	<0.001
Waistline (cm)	88.96 ± 10.45	87.52 ± 11.52	0.054	91.00 ± 9.33	86.08 ± 11.82	<0.001	90.57 ± 10.55	87.42 ± 11.21	<0.001	94.35 ± 9.74	86.90 ± 11.01	<0.001
Hip circumference (cm)	98.81 ± 6.58	98.46 ± 7.16	0.256	99.62 ± 6.54	97.89 ± 7.13	<0.001	99.75 ± 10.55	98.29 ± 7.03	0.002	101.28 ± 7.26	98.09 ± 6.78	<0.001
SBP (mmHg)	132.38 ± 18.53	128.11 ± 18.78	<0.001	132.48 ± 18.67	127.87 ± 18.65	<0.001	132.18 ± 19.02	129.09 ± 18.67	0.018	129.65 ± 16.80	129.73 ± 19.12	0.954
DBP (mmHg)	78.96 ± 11.57	76.21 ± 11.31	<0.001	79.39 ± 11.32	75.81 ± 11.38	<0.001	78.34 ± 11.08	76.96 ± 11.56	0.085	78.43 ± 10.93	77.03 ± 11.57	0.116
GLU (mmol/L)	5.50 ± 1.42	5.75 ± 1.97	0.005	5.86 ± 1.87	5.41 ± 1.81	<0.001	5.70 ± 1.86	5.57 ± 1.59	0.322	5.87 ± 1.82	5.54 ± 1.61	0.005
LDL-c (mmol/L)	3.45 ± 0.60	2.38 ± 0.55	<0.001	2.91 ± 0.81	2.70 ± 0.72	<0.001	3.86 ± 0.43	2.51 ± 0.56	<0.001	2.49 ± 0.81	2.84 ± 0.75	<0.001
HDL-c (mmol/L)	1.41 ± 0.33	1.30 ± 0.29	<0.001	1.21 ± 0.24	1.43 ± 0.32	<0.001	1.38 ± 0.29	1.33 ± 0.31	0.041	0.94 ± 0.08	1.42 ± 0.28	<0.001
TC (mmol/L)	5.86 ± 0.66	4.33 ± 0.60	<0.001	5.27 ± 1.00	4.67 ± 0.87	<0.001	6.10 ± 0.74	4.60 ± 0.77	<0.001	4.55 ± 1.09	4.98 ± 0.93	<0.001
TG (mmol/L)	1.52 ± 0.95	2.17 ± 1.41	<0.001	2.76 ± 1.29	1.09 ± 0.31	<0.001	1.88 ± 0.79	1.73 ± 1.27	<0.001	2.73 ± 1.93	1.59 ± 0.88	<0.001
ApoE ϵ4 (*n* (%))	53 (11.18)	98 (12.45)	0.531	49 (9.68)	102 (13.42)	0.051	27 (10.47)	124 (12.30)	0.453	23 (11.73)	127 (11.87)	1
Aβ42 (pg/mL)	50.32 ± 26.32	52.82 ± 27.93	0.527	55.07 ± 27.59	48.72 ± 27.44	0.002	50.84 ± 28.32	52.36 ± 27.46	0.994	59.29 ± 29.58	50.79 ± 26.55	0.003

Aβ, amyloid beta; BMI, body mass index; TC, total cholesterol; TG, triglyceride; HDL-c, high-density lipoprotein; LDL-c, low-density lipoprotein; SBP, systolic blood pressure; DBP, diastolic blood pressure; ApoE, apolipoprotein E. A subject with TC ≥ 5.18 mmol/L and/or TG ≥ 1.70 mmol/L and/or LDL-c ≥ 3.37 mmol/L and/or HDL-c ≤ 1.04 mmol/L is defined as dyslipidemia; A subject with at least one allele of ϵ4 is defined as ApoE ϵ4 carrier.

**Table 3:** Comparison of serum Aβ42 levels among participants divided by blood pressure.

	Normal blood pressure (*n* = 862)	Hypertension (*n* = 399)	*p*-value
Age (years)	48.083 ± 17.68	60.16 ± 14.32	<0.001
Male (*n* (%))	448 (51.97)	228 (57.14)	0.089
Smoking (*n* (%))	282 (32.71)	111 (27.82)	0.078
Drinking (*n* (%))	141 (16.36)	55 (13.78)	0.277
BMI (kg/m^2^)	25.97 ± 3.85	27.05 ± 3.71	<0.001
Waistline (cm)	86.91 ± 11.35	90.33 ± 10.29	<0.001
Hip circumference (cm)	98.35 ± 6.87	98.85 ± 7.09	0.073
SBP (mmHg)	119.96 ± 11.70	150.81 ± 12.98	<0.001
DBP (mmHg)	72.91 ± 8.23	86.61 ± 11.45	<0.001
GLU (mmol/L)	5.44 ± 1.48	5.93 ± 1.93	<0.001
LDL-c (mmol/L)	2.76 ± 0.78	2.84 ± 0.74	0.070
HDL-c (mmol/L)	1.33 ± 0.30	1.37 ± 0.33	0.041
TC (mmol/L)	4.85 ± 1.00	5.04 ± 0.90	0.001
TG (mmol/L)	1.71 ± 1.21	1.89 ± 1.13	0.011
ApoE ϵ4 (*n* (%))	97 (11.25)	54 (13.53)	0.263
Aβ42 (pg/mL)	50.49 ± 30.93	55.37 ± 33.96	0.164

Aβ, amyloid beta; BMI, body mass index; TC, total cholesterol; TG, triglyceride; HDL-c, high-density lipoprotein; LDL-c, low-density lipoprotein; SBP, systolic blood pressure; DBP, diastolic blood pressure; ApoE, apolipoprotein E. A subject with systolic blood pressure (SBP) ≥ 140 mmHg and/or diastolic blood pressure (DBP) ≥ 90 mmHg is defined as hypertension. A subject with at least one allele of ϵ4 is defined as an ApoE ϵ4 carrier.

**Table 4:** Multiple linear regression analyses for the exploration of the potential correlations between serum Aβ42 levels and BMI, blood lipids, and blood pressure.

Model	Covariate	Aβ42 (pg/mL)		
		*β*	95% CI	*p*-value
1	BMI	2.694	0.386 to 5.002	0.022
	HDL-c	−1.605	−3.461 to 1.252	0.085
	TC	−1.005	−5.041 to 3.030	0.625
	TG	−0.391	−4.940 to 4.159	0.866
	SBP	−0.300	−0.652 to 0.052	0.094
	DBP	0.541	0.016 to 1.066	0.043
2	BMI	2.703	0.395 to 5.010	0.023
	LDL-c	−2.552	−8.281 to 3.177	0.381
	HDL-c	−1.741	−3.428 to −0.549	0.043
	TG	−0.984	−5.025 to 3.056	0.633
	SBP	−0.301	−0.625 to 0.051	0.094
	DBP	0.546	0.021 to 1.071	0.042

Model 1 was adjusted for gender, age, smoking, drinking, waistline and lip circumference, BMI, TC, TG, HDL-c, SBP, DBP, GLU, and ApoE genotypes. Model 2 was adjusted for sex, age, smoking, drinking, waistline and lip circumference, BMI, TG, LDL-c, HDL-c, SBP, DBP, GLU, and ApoE genotypes. β, the unstandardized regression coefficient; Aβ, amyloid beta; BMI, body mass index; TC, total cholesterol; TG, triglyceride; HDL-c, high-density lipoprotein; LDL-c, low-density lipoprotein; SBP, systolic blood pressure; DBP, diastolic blood pressure; ApoE, apolipoprotein E.

**Table 5:** Comparison of serum Aβ42 levels among participants associated with MetS, divided by age.

	Young (≥ 18 and ≤ 29) (*n* = 115)	*p*-value	Middle (≥ 30 and ≤ 64) (*n* = 853)	*p*-value	Elder (≥ 65 and ≤ 79) (*n* = 244)	*p*-value
Age (years)	25.63 ± 2.33		48.07 ± 9.28		71.17 ± 4.36	
Male (*n* (%))	68 (59.1%)		383 (44.9%)		114 (46.7%)	
Smoking (*n* (%))	51 (44.3%)		258 (30.2%)		69 (28.3%)	
Drinking (*n* (%))	21 (18.3%)		128 (15.0%)		21 (8.6%)	
ApoE ϵ4 (*n* (%))	10 (8.7%)		110 (12.9%)		25 (10.2%)	
MetS = 1	−0.2 (−28.1, 38.7)	0.99	22.4 (−0.3, 50.1)	0.05	174.2 (53.7, 388.9)	<0.001
MetS = 2	66.1 (15.3, 139.3)	0.007	60.8 (31.7, 96.3)	<0.001	174.5 (55.4, 384.9)	<0.001
MetS = 3	100.6 (35.3, 197.4)	<0.001	59.9 (29.8, 96.9)	<0.001	240.4 (95.6, 492.4)	<0.001
MetS = 4	151.0 (12.3, 461.0)	0.03	48.6 (17.3, 88.1)	0.001	230.9 (83.4, 497.1)	<0.001
MetS = 5	70.9 (−56.1, 565.6)	0.44	49.4 (4.0, 114.7)	0.03	204.6 (45.1, 539.6)	0.003
MetS ≥ 3	105.9 (43.9, 194.6)	<0.001	55.3 (28.0, 88.3)	<0.001	233.5 (96.1, 467.1)	<0.001
Overweight (kg/m^2^) (24–27.9)	56.8 (11.3, 120.8)	0.01	44.1 (23.6, 68.0)	<0.001	183.0 (109.7, 281.8)	<0.001
Obesity (kg/m^2^) (28–41.9)	116.9 (61.4, 191.6)	<0.001	76.6 (51.8, 105.4)	<0.001	112.3 (57.9, 185.5)	<0.001
Waist/hip ratio (>0.9 for men, >0.8 for women)	71.2 (25.6, 133.3)	<0.001	37.1 (21.0, 55.3)	<0.001	64.3 (19.5, 125.9)	0.002
Hypertension (SBP ≥140, DBP ≥90)	−33.8 (−68.9, 41.0)	0.28	8.1 (−5.5, 23.6)	0.26	10.5 (−15.0, 43.8)	0.45
GLU (6.01–6.98 mmol/L)	0.0 (0.0, 0.0)		−0.8 (−18.5, 20.7)	0.93	33.5 (−6.9, 91.4)	0.12
GLU (7–22 mmol/L)	−32.1 (−84.2, 191.2)	0.6	14.7 (−8.6, 43.9)	0.24	−7.4 (−35.2, 32.4)	0.67

MetS = 1, one factor; MetS = 2, two out five factors; MetS = 3, three out five factors; MetS = 4, four out five factors; MetS = 5, all five factors; MetS ≥ 3, at least three factors.

### 3.2 Increased serum Aβ42 levels are associated with obesity

**Figure 2:**
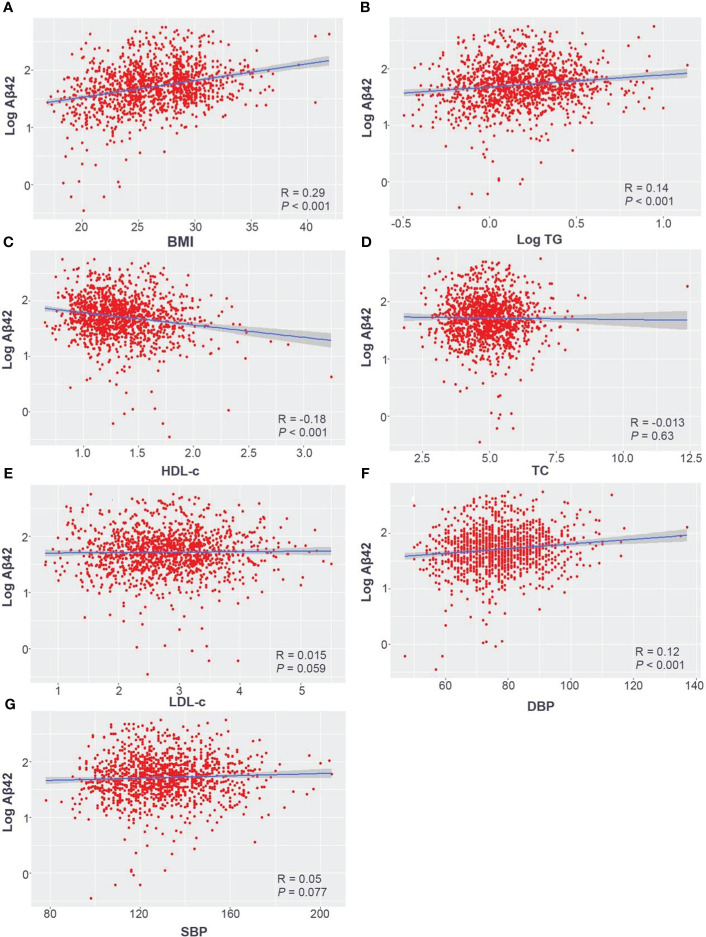
Pearson correlation analysis of log-transformed serum Aβ42 levels with the BMI **(A)**, log-transformed TG **(B)**, HDL-c **(C)**, TC **(D)**, LDL-c **(E)**, DBP **(F)**, and SBP **(G)** in the Han Chinese population. Aβ42, amyloid beta 42; BMI, body mass index; TC, total cholesterol; TG, triglyceride; HDL-c, high-density lipoprotein; LDL-c, low-density lipoprotein; SBP, systolic blood pressure; DBP, diastolic blood pressure.

The participants were divided into three groups according to their BMI, and serum Aβ42 levels were significantly different between the normal weight, overweight, and obesity groups (**Table 1**). Pearson correlation analysis demonstrated a positive linear correlation between BMI and log-transformed serum Aβ42 levels in participants of all groups (*R* = 0.29; *p* < 0.001) (**Figure 2A**). Subsequently, multiple linear regression analysis was carried out to further elucidate the correlation between serum Aβ42 level and BMI. All covariates were not colinear, except for TC and LDL-c; hence, two models were built to avoid their cross-interference (**Table 2**). After adjusting for confounding factors, both models showed that serum Aβ42 levels were positively correlated with BMI, with the unstandardized regression coefficient (*β*) = 2.694, *p* < 0.05 in model 1 and *β* = 2.703, *p* < 0.05 in model 2 (**Table 4**). These results elucidated a positive linear correlation trend between serum Aβ42 levels and BMI in a Han Chinese population for the first time.

### 3.3 Serum Aβ42 levels are correlated to concentrations of lipid metabolism biomarkers

All subjects were divided into different groups according to TC, TG, LDL-c, and HDL-c levels. There were no significant differences in serum Aβ42 levels between normal TC and high TC groups or between normal LDL-c and high LDL-c groups (**Table 2**). Meanwhile, serum Aβ42 levels were significantly higher in the low HDL-c group than the normal HDL-c group (*p* = 0.003) and higher in the high TG group compared to the normal TG group (*p* = 0.002) (**Table 2**). In addition, the correlations of TC, LDL-c, HDL-c, and log-transformed TG with log-transformed serum Aβ42 levels in participants of both groups were further analyzed by Pearson correlation analysis. The results showed that serum Aβ42 levels were positively correlated with the TG level (*R* = 0.14; *p* < 0.001) (**Figure 2B**) and negatively correlated with the HDL-c level (*R* = −0.18; *p* < 0.001) (**Figure 2C**). The TC and LDL-c levels showed no linear correlations with serum Aβ42 levels (**Figures 2D, E**). To further explore the relationship between serum Aβ42 and blood lipids, the two multiple regression models described in the previous section were applied to elucidate the linear correlations between TG and HDL-c levels and serum Aβ42 levels. The results showed that after adjusting for confounding factors, serum Aβ42 levels were negatively and independently correlated with the HDL-c level in participants of both the normal blood lipid group and dyslipidemia group in model 2 (*β* = −1.741; *p* < 0.05) (**Table 4**).

### 3.4 Association between high serum Aβ42 level and hypertension

Serum Aβ42 levels were compared between the normal blood pressure group and the hypertension group, and no significant difference in serum Aβ42 levels was found between these two groups by Mann–Whitney *U* test (50.49 pg/mL vs. 55.37 pg/mL, *p* = 0.164). The Pearson correlation analysis showed that serum Aβ42 levels were positively correlated with DBP in participants of both groups (*R* = 0.12, *p* < 0.001, **Figure 2F**), while not correlated with SBP (**Figure 2G**). After adjusting for confounding factors, multiple regression analyses further demonstrated the positive correlations between serum Aβ42 levels and DBP in both models (model 1: *β* = 0.541; *p* < 0.05; model 2: *β* = 0.546; *p* < 0.05) (**Table 4**).

### 3.5 Correlation between serum Aβ42 levels and MetS in adults

To determine which out of the five MetS components, including abdominal obesity, high glucose, high triglyceride, low/high-density lipoprotein cholesterol levels, and hypertension predominantly cause Aβ42 elevations in the peripheral blood, we analyzed correlations between serum Aβ42 of participants with individual criteria of the metabolic syndrome at least three abnormal findings out of five factors. According to the above analyses, the level of serum Aβ42 was significantly higher with the presence of abdominal obesity, high TC, low HDL-c, and hypertension, but not impaired fasting glucose (**Tables 1**–**5**).

To evaluate the association between Aβ42 and metabolic syndrome, multivariate logistic regression analyses were performed (**Table 5**). Metabolic syndrome was treated as the outcome measurement. After multivariate adjustment including age, gender, smoking, alcohol, and ApoE carrier, logistic regression analysis showed a significant association between Aβ42 level and metabolic syndrome (OR, 26.8; 95% CI, 14.2–40.7; *p* < 0.001) (**Table 6**).

**Table 6:** Comparison of serum Aβ42 levels among participants associated with MetS.

	Age 18 to 79 (*n* = 1,212)	*p*-value
MetS = 1	38.8 (17.1, 64.5)	<0.001
MetS = 2	79.3 (51.8, 111.9)	<0.001
MetS = 3	87.9 (58.1, 123.2)	<0.001
MetS = 4	80.0 (47.5, 119.6)	<0.001
MetS = 5	78.9 (32.3, 141.8)	<0.001
MetS ≥ 3	26.8 (14.2, 40.7)	<0.001

MetS = 1, one factor; MetS = 2, two out five factors; MetS = 3, three out five factors; MetS = 4, four out five factors; MetS = 5, all five factors; MetS ≥ 3, at least three factors.

To determine whether the level of Aβ42 in peripheral blood corresponds to the number of clinical measures fulfilling MetS criteria, we further categorized participants into five groups (MetS1–MetS5). The serum Aβ42 levels significantly increased with the number of MetS criteria fulfilled from one to five (**Tables 5**, **6**), which was also consistent with the previous individual analysis of the five factors. In addition, as expected, we found that a high serum Aβ42 level was related to MetS.

## 4 Discussion

This clinical observational study demonstrated that Aβ42 levels are positively correlated with BMI and DBP and negatively correlated with levels of HDL-c. Moreover, we uncovered positive associations between serum Aβ42 levels and MetS, along with their individual clinical measures. We found no significant relationships between peripheral Aβ42 levels and TC, TG, or LDL-c. Our study contributes to a body of evidence that attempts to explain the direct role of serum Aβ24 in metabolic conditions by adding unique data from the Han Chinese population, which has been understudied in this field, along with strong statistical analyses of a large sample size.

Previous clinical studies in various populations have demonstrated similar positive correlations between Aβ42 levels and MetS and related risk factors. Higher levels of Aβ42 in the peripheral blood of adults and children were associated with higher BMI (29, 41). Using cross-sectional analysis, Wei et al. (42) reported that plasma Aβ24 levels in 1,436 adults were positively correlated with HDL-c and negatively correlated with TG. Another 5-year prospective study in 440 elderly persons showed that participants with the highest third of TC or LDL-c at baseline showed lower plasma Aβ42 levels at 5 years (31). However, there has also been contradictory evidence regarding the relationship between Aβ42 and metabolic disorders. A study reported a negative and marginal correlation (*p* = 0.05) between BMI and serum Aβ42 levels in 530 elderly African American, Caribbean Hispanic, and White participants (43). These opposing conclusions could be a result of differences in participant ethnic groups and/or immunodetection methods, including the use of different polyclonal antibodies.

Several mechanisms have been proposed to explain the correlations observed between metabolic abnormalities, serum Aβ42 levels, and AD. Circulating HDL-c has been shown to play an important role in translocating Aβ42 for degradation and/or excretion (44). As such, low levels of HDL-c may reflect worse conditions for solubility and contribute to the imbalance of Aβ42 degradation. Indeed, our study demonstrated a significant and negative correlation between HDL-c and Aβ42 levels. Furthermore, accumulating evidence suggests that elevated midlife blood pressure is associated with an increased risk of cognitive impairment and dementia (45, 46), and thus blood pressure should be considered a potential confound for serum Aβ42 levels (47, 48). Unsurprisingly, our results showed a positive correlation between DBP and Aβ42 levels, consistent with previous literature (49). This can be explained mechanistically, as elevated serum Aβ42 has been shown to reduce endothelial NO synthase, leading to lower NO production, impaired vascular relaxation, and elevated blood pressure (50). Controlled animal studies also support human observational studies. Under a high-fat diet, endogenous melatonin reduction (EMR) mice showed decreased anti-stress ability and had greater body weight and more obvious hepatic steatosis compared with the wild-type group; furthermore, 8-month-old EMR mice had AD-like phenotypes, including Iba-1 activation, Aβ protein deposition, and decreased spatial memory ability (51).

Yet another pathway by which metabolic disturbances can affect Aβ42 levels is through neuroinflammation and blood–brain-barrier (BBB) disruption. Previous studies show that MetS is associated with increased levels of reactive oxygen species, glucose, fibrinogen, and free fatty acids from the vasculature, skeletal muscle, liver, and adipose tissue, prompting insulin resistance (IR), hyperglycemia, inflammation, and dyslipidemia, respectively (52). General inflammation, neuroinflammation from IR, and the increase in inflammatory marker IL-6 can upregulate the expression of APP (53), which is expressed in both central and peripheral tissues and is cleaved by proteases to generate Aβ. Aβ can cross the BBB to form a dynamic equilibrium in the CSF and peripheral blood. LRP1 on the BBB is responsible for transporting Aβ from the CSF to the periphery, while receptors for advanced glycation end products (RAGE) can transport peripheral Aβ into the brain parenchyma (54). The expression levels of LRP1 and RAGE on the BBB are related to the risk of AD. Studies have shown that elevated Aβ can reduce the expression of LRP1 and increase the expression of RAGE levels (55). Thus, in the presence of MetS, serum Aβ42 rises with general inflammation and neuroinflammation, leading to Aβ accumulation in the brain and an unbalanced equilibrium favoring Aβ transport into the CSF by RAGE, resulting in a cycle that aggravates Aβ deposition in the brain.

While serum Aβ42 has been proposed as a biomarker for monitoring the systemic risk of AD in mid-life to predict AD occurrence in late life, published results on the correlation of blood Aβ and CSF Aβ with the presence of AD are inconsistent and even contradictory to date. Factors such as liver function (56–58) and, as we and others have demonstrated, a host of metabolic abnormalities, greatly affect levels of Aβ in the periphery. Aβ in the periphery is not only attributed to efflux of the brain Aβ but is also derived from the proteolytic cleavage of APP expressed in peripheral organs and tissues (8, 59–62). Hence, serum APP and Aβ42 levels may not reflect the levels of APP and Aβ42 in the brain because a large amount of plasma Aβ comes from peripheral sources (4, 63–65). In contrast, we have demonstrated positive correlations between serum Aβ42 levels and MetS, along with each of its associated factors. Furthermore, we found that the serum Aβ42 levels were significantly increased with the number of MetS criteria fulfilled. Thus, we propose the potential role of serum Aβ42 as a direct biomarker for MetS rather than for AD.

Aside from studies on Aβ, prior literature has more generally examined the biological complexity of AD pathophysiology through a systemic lens. For example, studies have shown that the selective disruption of circadian timing within cortical and limbic circuits underlies certain cognitive deficits in AD, and events in AD pathogenesis including amyloid deposition, oxidative stress, and cell death in turn lead to further disruption of the circadian rhythm (66, 67). Circadian rhythm disruption has also been related to a lack of hormonal homeostasis and nonalcoholic fatty liver disease (68, 69) and may be a major contributor to key components of MetS and its comorbidities (70). The circadian system could be a possible link between the metabolic disturbances we observe associated with AD, meriting further study.

It is clear that AD and MetS are complex systemic diseases with countless involved pathways and numerous methods of study. Here, we have presented data that reaffirms the connection between Aβ42 and MetS and supports the role of Aβ42 as a clinical biomarker for MetS. We also acknowledge the following limitations of our work. Firstly, our study population includes only the Han population and precludes conclusions that span multiple ethnic groups, particularly given the contradicting evidence from previous literature. Secondly, our study takes place at a single hospital, which may limit its generalization to other locations. A multicenter study utilizing our published protocols would greatly strengthen the power of the results. Finally, we believe that the addition of CSF Aβ42 measurements, while logistically difficult, would greatly inform our understanding of the mechanistic connection between MetS and AD by correlating brain and serum Aβ42 levels. We plan to collect CSF specimens in a future study for this investigation.

## 5 Conclusions

To the best of our knowledge, this is the first comprehensive report on the correlations of serum Aβ42 levels with BMI, blood lipids, blood pressure, and MetS in the Han Chinese population in Southwest China. We reveal strong correlations between serum Aβ42 levels and MetS, as well as the individual factors comprising MetS. Furthermore, our study found that BMI and DBP levels were positively associated with serum Aβ42 levels, while HDL-c was negatively associated with serum Aβ42 levels. These results indicate that dysregulated MetS is associated with higher serum Aβ42 levels. Therefore, we recommend serum Aβ42 be used as a direct risk biomarker for MetS and its components rather than for AD. The study was exploratory and aimed to contribute to the body of controversial evidence surrounding Aβ42. The findings encourage further research investigating the detailed mechanisms of how serum Aβ42 levels interact with brain Aβ42 levels in MetS and AD.

## Data availability statement

The original contributions presented in the study are included in the article/supplementary material. Further inquiries can be directed to the corresponding authors.

## Ethics statement

The studies involving humans were approved by Ethics Committee of Sichuan Academy of Medical Sciences & Sichuan Provincial People’s Hospital (2020No.281). The studies were conducted in accordance with the local legislation and institutional requirements. The participants provided their written informed consent to participate in this study.

## Author contributions

KL: Data curation, Formal analysis, Writing – original draft. XZ: Writing – original draft, Writing – review & editing, Formal analysis. YRL: Data curation. DL: Data curation. YYL: Data curation. TZ: Data curation. CF: Data curation. LL: Formal analysis, Writing – original draft. YH: Writing – review & editing. LJ: Conceptualization, Funding acquisition, Project administration, Writing – review & editing.
